# Electrophysiological assessment of temporal envelope processing in cochlear implant users

**DOI:** 10.1038/s41598-020-72235-9

**Published:** 2020-09-21

**Authors:** Robin Gransier, Robert P. Carlyon, Jan Wouters

**Affiliations:** 1 Department of Neurosciences, KU Leuven, ExpORL, Herestraat 49, Box 721, 3000 Leuven, Belgium; 2 Cambridge Hearing Group, MRC Cognition and Brain Sciences Unit, University of Cambridge, 15 Chaucer Road, Cambridge, CB2 7EF UK

**Keywords:** Sensory processing, Neuroscience, Auditory system, Cortex, Midbrain

## Abstract

Cochlear-implant (CI) users rely on temporal envelope modulations (TEMs) to understand speech, and clinical outcomes depend on the accuracy with which these TEMs are encoded by the electrically-stimulated neural ensembles. Non-invasive EEG measures of this encoding could help clinicians identify and disable electrodes that evoke poor neural responses so as to improve CI outcomes. However, recording EEG during CI stimulation reveals huge stimulation artifacts that are up to orders of magnitude larger than the neural response. Here we used a custom-built EEG system having an exceptionally high sample rate to accurately measure the artefact, which we then removed using linear interpolation so as to reveal the neural response during continuous electrical stimulation. In ten adult CI users, we measured the 40-Hz electrically evoked auditory steady-state response (eASSR) and electrically evoked auditory change complex (eACC) to amplitude-modulated 900-pulses-per-second pulse trains, stimulated in monopolar mode (i.e. the clinical default), and at different modulation depths. We successfully measured artifact-free 40-Hz eASSRs and eACCs. Moreover, we found that the 40-Hz eASSR, in contrast to the eACC, showed substantial responses even at shallow modulation depths. We argue that the 40-Hz eASSR is a clinically feasible objective measure to assess TEM encoding in CI users.

## Introduction

Electrical stimulation of the auditory nerve, by means of a cochlear implant (CI), restores hearing to severely-hearing impaired people^1^. CIs are especially successful when listening to speech in quiet. However, performance with a CI deteriorates markedly when listening conditions become more challenging, e.g. speech in noise. Speech intelligibility in challenging listening situations depends heavily on the speech features available to the CI user. CI users do well in quiet listening conditions, since only a limited number of spectral channels are required to perceive speech intelligibly as long as the temporal envelope modulations (TEMs) of speech are preserved^2,3^. Conventional CI stimulation strategies are based on this phenomenon and predominately transmit the TEMs of the received acoustical signal to the auditory nerve by means of a limited number of spectral channels^4^. Conventional CIs have between 12 to 24 stimulation electrodes but, due to current spread within the cochlea, listeners only have access to 8 to 10 independent channels of information^5–8^. Some CI users, however, benefit from more spectral channels when facing more challenging listening conditions^9,10^.

The ability of the stimulated neural ensembles to encode the TEMs is, apart from the perceptually unique spectral channels available to the CI user, important for speech perception^11^. Studies of behavioral and electrophysiological assessments of TEM encoding show that a uniformly poor or highly variable TEM encoding across the stimulated neural ensembles is associated with degraded speech intelligibility in noise^12,13^. Furthermore, disabling or adjusting stimulation parameters of the CI electrodes that stimulate poor TEM encoding neural ensembles can positively affect speech perception in noise^12,14,15^. These findings suggest that CI users could benefit from methods that disable or re-program subsets of electrodes based on the TEM encoding abilities of the stimulated neural ensembles^12,14^.

For the clinical assessment of TEM encoding, reliable and time-efficient measures are needed. Objective assessment of the TEM encoding has its advantage over behavioral methods/metrics, such as the often-used modulation detection thresholds (MDTs), as they do not rely on the active cooperation of the CI recipient. Furthermore, they can be used to evaluate modulation encoding at the supra-threshold modulation depths that are important for speech perception. Potential objective measures are the electrically-evoked 40-Hz auditory steady-state response (eASSR) and the electrically-evoked auditory change complex (eACC). Both can be measured by means of non-invasive EEG techniques.

The 40-Hz eASSR is a phase-locked neural response to the temporal envelope of a 40-Hz modulated stimulus^16^ and originates from multiple sources across the auditory pathway; including the brainstem, thalamus and auditory cortex^17,18^. The 40-Hz eASSR is measurable in most adult CI users^13,19^, its magnitude correlates well with MDTs^20^, and it can capture the variability in TEM processing across the stimulated neural ensembles which is in turn associated with speech-perception-in-noise outcome^13^. Moreover, its acoustic analog, the ASSR, is used clinically to assess the frequency specific hearing thresholds in infants with a suspicion of hearing loss^21^. Measuring eASSRs in CI users is, however, extremely challenging due to the stimulation artifacts that share the same frequency characteristics as the eASSR^19,22,23^. This makes it difficult, without proper removal, to distinguish between a true neural response and the stimulation artifact.

**Figure 1:**
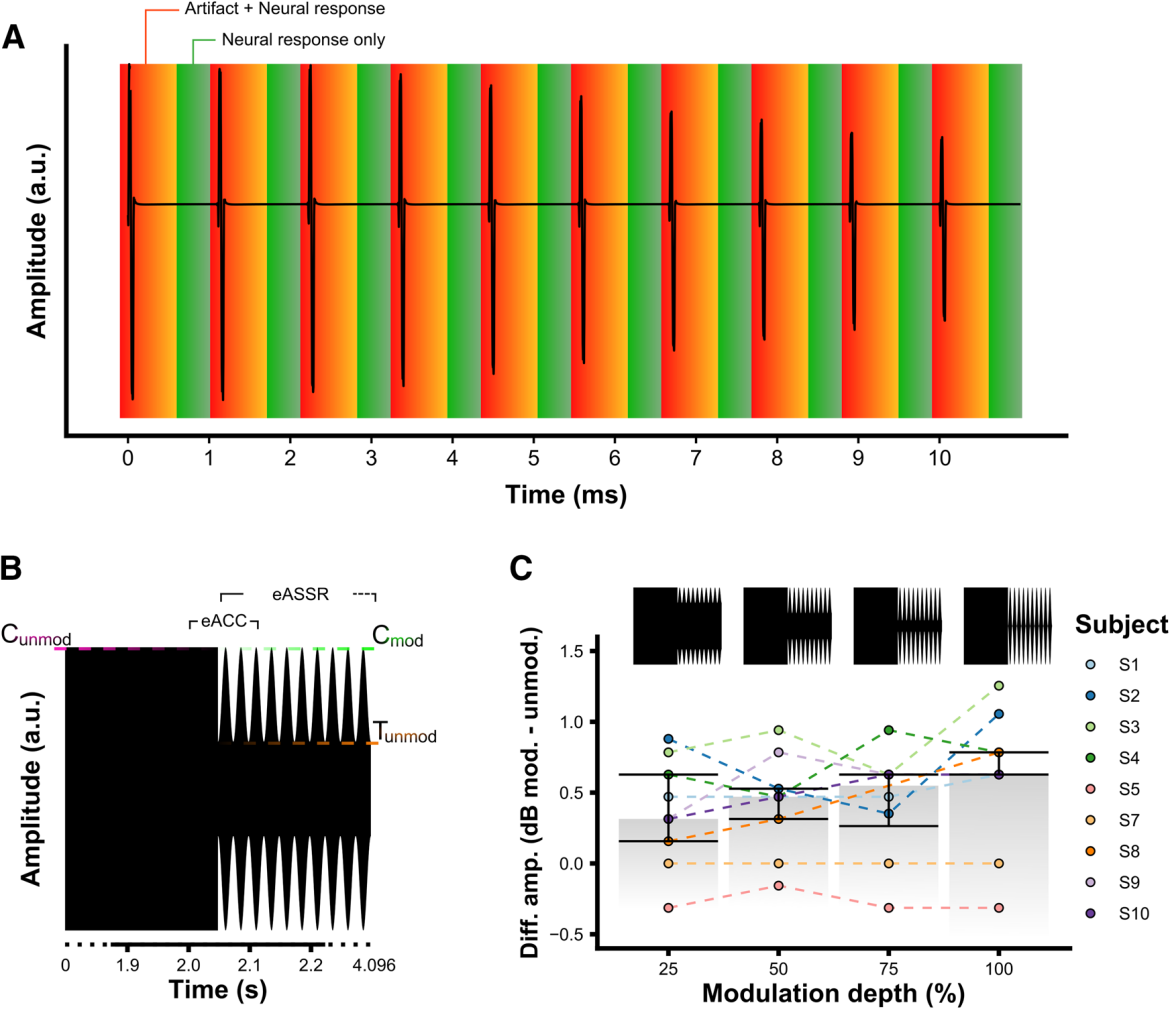
Method illustrations and behavioral results. (**A**) Illustration of linear interpolation between artifact free samples before and after the stimulation artifact. The artifact is removed by linearly interpolating between the artifact free regions. (**B**) Illustration of a single trial used in the EEG experiments to assess the effect of modulation depth on the electrically-evoked auditory change complex (eACC) and the 40-Hz electrically-evoked auditory steady-state response (eASSR). A single trial consists of a 2.048 s unmodulated pulse train followed by a 2.048 s modulated pulse train. This illustration is an example of a 100% modulation where C_unmod_ and C_mod_ are equal current level and the minima of the modulation are set to T_unmod_. (**C**) The difference in current required to obtain an equal loudness percept between the unmodulated and modulated stimuli as a function of modulation depth. Bars show the group average, and the error bars ± one quantile. The different stimuli on top are for illustrative purposes in which T_unmod_ is set to zero. Figures are made in R^69^.

Although several artifact removal methods have been reported in the literature to record eASSRs free from stimulation artifacts in CI users^24–27^, only linear interpolation between artifact free samples before and after the stimulation artifact (Fig. 1A) has resulted in consistent success^13,19,22,28,29^. Linear interpolation, however, can only be successfully applied if the artifact duration is shorter than the inter-pulse interval of the stimulation sequence. For pulse trains stimulated in monopolar mode and at clinically relevant stimulation rates, which are typically ≥ 500 pulses per second (pps), this is often not the case^23^. The maximum pulse rate reported in the literature for which linear interpolation is applicable is 500 pps^13,19,29^. One factor that affects the CI artifact duration is the sampling rate and the inherent anti-aliasing filter applied to record the EEG^30^. When using conventional EEG-recording this results in a distorted artifact waveform and an artifact duration that exceeds the inter-pulse intervals of clinically relevant pulse rates.

One potential alternative objective assessment of TEM encoding that is less troubled by the stimulation artifact is the ACC. The ACC is a transient cortical response to a change in stimulus—e.g., from unmodulated to modulated—that originates from the auditory cortex^31,32^. In the following we refer to the ACC when it is evoked in the acoustically stimulated auditory pathway and eACC when it is evoked in the electrically stimulated auditory pathway. In normal-hearing listeners the ACC strength is related to modulation detection^32^. Given the cortical origin of the ACC it can potentially function as a final detector of how well the TEMs are encoded in the neural signal. The ACC has been successfully used as an electrophysiological measure of gap detection^33^, frequency change detection^34^, and the eACC for electrode discrimination^35–37^ in CI users. Furthermore, the eACC N1 amplitude shows a similar pattern as the temporal modulation transfer function of CI users^38^. It is, however, unclear if the eACC is able to reflect the TEM encoding ability of the stimulated neural ensembles of an individual CI user.

Here we investigate how the 40-Hz eASSR and the eACC reflect TEM encoding in the auditory pathway of ten adult CI users. We use different percentages of modulation depths (MD_%_) to model the various degrees of TEM encoding at the level of the stimulated neural ensembles, which are known to vary across CI users and stimulation sites^12,13,39^. We hypothesize that both the 40-Hz eASSR and the eACC are differently affected by modulation depth, due the different origins of the two responses in the ascending auditory pathway. Furthermore, we use a hyper-rate sampling EEG system, which was specially designed—to our specifications—to limit the distortion of the artifact waveform and therefore potentially shorten the stimulation artifact duration. We hypothesize that this approach enables the removal of the stimulation artifacts when measuring neural responses from CI users, especially eASSRs, to clinically relevant stimulation rates and in monopolar mode.

## Results

Ten adult CI users took part (mean age = 56.1 years, 9 female); all had a history of long-term hearing impairment with an average CI use of 4.3 years (see “Methods” section). The study consisted of two intertwined EEG experiments. We used, in all experiments a stimulus that is representative of those used in conventional CI systems, namely a train of symmetric biphasic pulses delivered in monopolar mode at a pulse rate of 900 pulses per second (pps). First, the effectiveness of CI artifact removal was assessed. Second, the effect of MD% on the eASSR and the eACC was investigated. In the first experiment we only used amplitude modulated pulse trains so as to measure the effectiveness of artifact removal on the measurement of eASSRs. The second experiment used a stimulus consisting of a constant pulse train immediately followed by a modulated pulse train (i.e., a single trial), and measured the eACC to the change in modulation; this also allowed us to measure an eASSR to the modulated portions. The effect of MD_%_ on the electrophysiological responses was investigated by changing the MD_%_ of the modulated part of the stimulus from 100 to 25% across conditions. Figure 1B shows an illustration of a single stimulation trial used to assess the effect of MD_%_ on the eACC and 40-Hz eASSR.

### Behavioral assessment of stimulation levels

Behavioral thresholds, comfort levels, and equal-loudness levels were assessed prior to each EEG experiment. This was done to create subject-appropriate stimuli and to ensure that any eACC to the transition between modulated and unmodulated portions was not attributable to long-term loudness differences between them^40,41^. Behavioral thresholds, comfort levels and the current needed for each condition to obtain an equal loudness perception across conditions and subjects, were assessed prior to the EEG experiments (see “Methods” section). Threshold (T_unmod_) and comfort (C_unmod_) levels for the unmodulated pulse train were, on average across subjects, 45.1 dB re 1µA (range 39.3 to 51.8 dB re 1µA) and 51 dB re 1µA (range 46.4 to 56.1 dB re 1µA), respectively. T_unmod_ served as the minima of the 100% amplitude modulated pulse train. The comfort levels of the 100% AM pulse train (C_mod_) were, on average across subjects, 51.3 dB re 1µA (range 47.1 to 56.1 dB re 1µA). The different MDs_%_ were based on the percentage of the dynamic range (dB current) between T_unmod_ and C_mod_, and loudness balancing was used to ensure that the ACC was evoked purely based on the difference in MD and not due to the difference in long-term loudness between the unmodulated and the modulated parts of a trial. During the loudness-balancing procedure (see “Methods” section), C_mod_ was loudness-balanced to C_unmod_ while the MD was kept fixed. Although there were individual differences in the C_mod_ compared to C_unmod_ (Fig. 1C), there was no significant group level effect of MD_%_ (i.e. 0 to 100%) on the current needed to obtain equal loudness as determined by a one-way ANOVA (F(4,39) = 0.055 p = 0.994).

### Artifact removal effectiveness for measuring ASSRs

**Figure 2:**
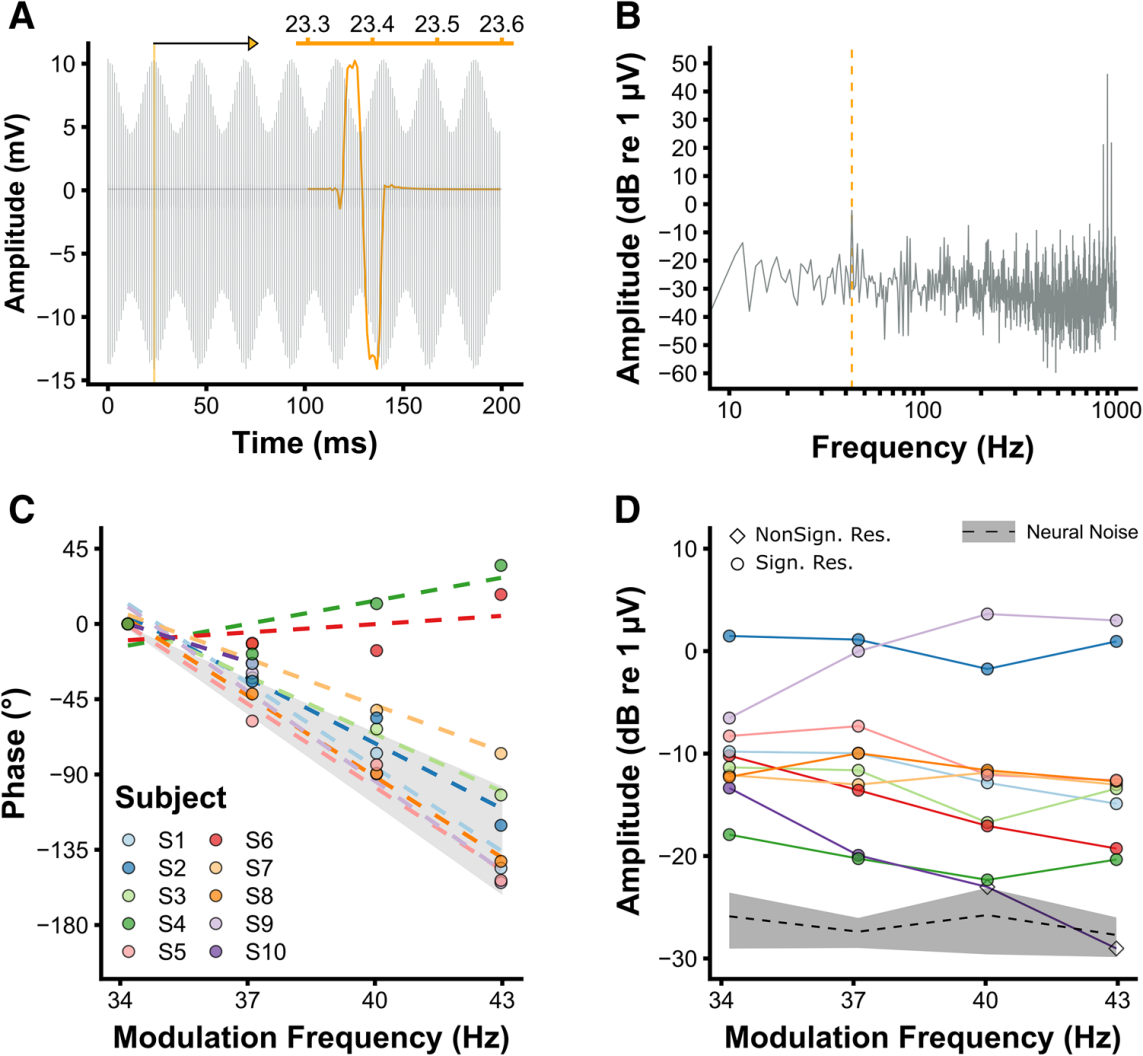
Artifact removal evaluation based on the eASSR. (**A**) The time-domain representation of an averaged epoch of subject S10 recorded from P10 (ipsilateral to the CI) referenced to Cz and without any artifact removal, i.e. the stimulation artifact. Only a segment of the epoch is showed for illustrative purposes. The modulation frequency was 43 Hz and the perceptual modulation depth was 100%. The orange waveform correspond to a zoomed-in single pulse between 23.3 and 23.6 ms. (**B**) The frequency-domain of the stimulation artifact as displayed in (**A**) (duration = 1.024 s, N = 285). (**C**) Each individual’s electrically-evoked auditory steady-state response (eASSR) phase after artifact removal as a function of modulation frequency, recorded from the recording electrode contralateral to the CI (P9 or P10). The offset of the phase is corrected so that the response phase of each subject at 34 Hz is zero. Only eASSRs that differed significantly from the noise are shown. The shaded area represents the range of phase slopes (30 to 50 ms) for normal-hearing listeners and CI users reported in the literature. (**D**) Each individual’s eASSR amplitudes as a function of modulation frequency. eASSRs that differed significantly from the neural background noise are shown in circles whereas those that were not significantly different from the neural background noise are shown in diamonds. The dashed black line and shaded gray area show the group’s average neural background noise ± one standard deviation. Data shown in (**C**,**D**) are recorded from the mastoid contralateral to the CI (P9 or P10) and referenced to Cz (N = 10). Figures are made in R^69^.

Figure 2A shows the stimulation artifacts of a representative subject in the time domain when no artifact removal is applied. Due to the asymmetric artifact waveform the artifact also contains a component at the response frequency (Fig. 2B) which leads, if not properly removed, into a false-positive detection of an ASSR.

Artifact removal effectiveness was assessed based on the phase characteristics of the measured response. We measured in addition to the 40-Hz eASSR, eASSRs to three additional frequencies (34, 37, and 43 Hz) and with the same stimulation parameters as the 100% MD stimulus, i.e. stimulated at a maximum perceptual modulation depth (MD_%_ = 100%) and loudness level. These are modulation frequencies have been shown to evoke ASSRs and eASSRs that originate from the same generator(s) as the 40-Hz (e)ASSR both in normal-hearing listeners^42,43^ and in CI users^19,22^. The rationale of using the phase characteristics to assess the effectiveness of artifact removal is based on the fact that the latency of the eASSR generator (i.e. the group delay) is identical to the phase slope (i.e. gradient of phase delay) when there is a linear relationship in phase across modulation frequencies. When using closely neighboring modulation frequencies to assess the phase slope, this slope will change accordingly to the latency of the generator(s) that is involved in generating the measured response^16,44^. The latency of the 34 to 43 Hz eASSR in CI users is around 40 ms^13,19^. However, if the recording is dominated by the stimulation artifacts the slope will be flat (i.e. a group delay of 0 ms), hence the recorded response does not reflect a neural response but that of the CI stimulation artifact.

**Figure 3:**
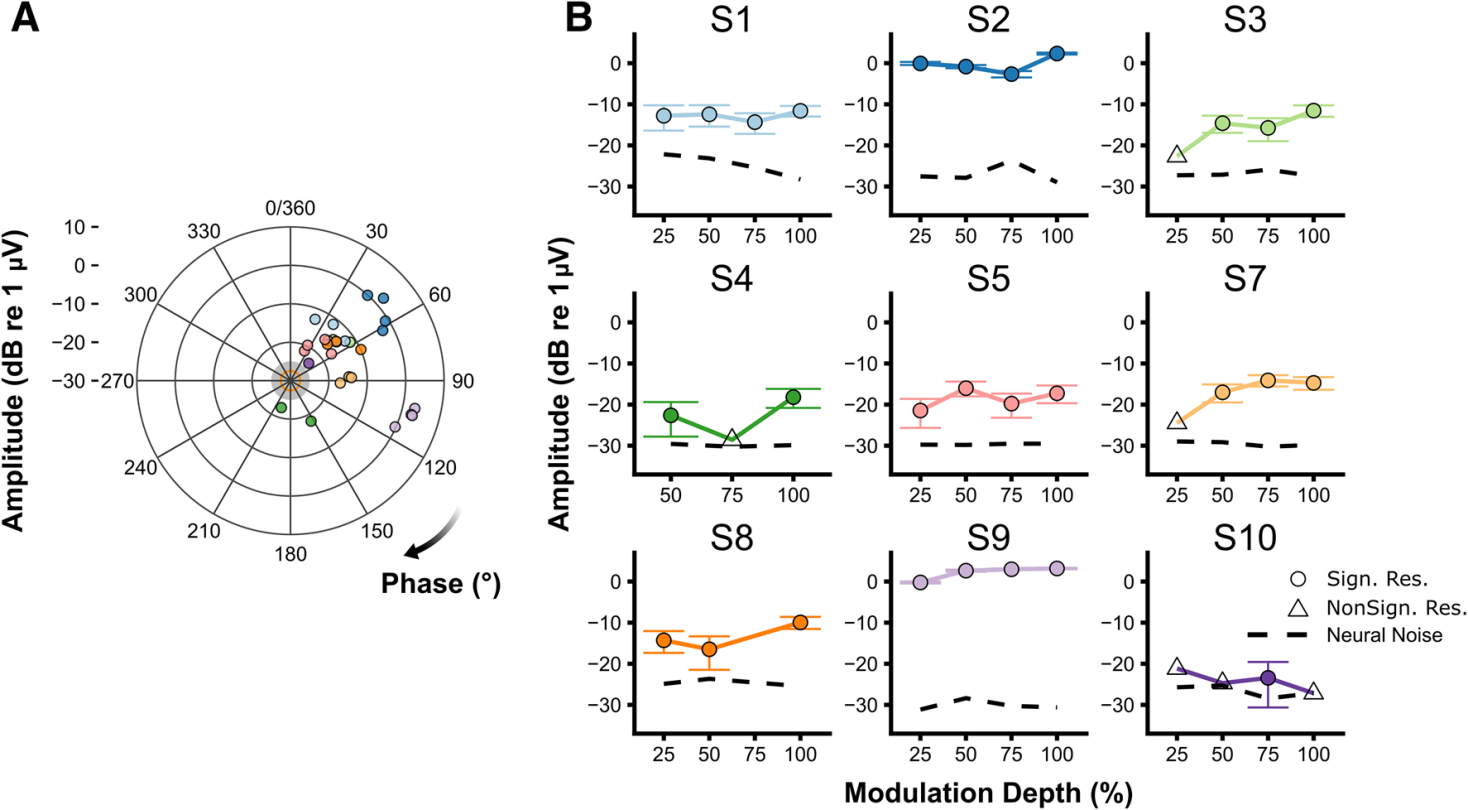
Effect of modulation depth on the 40-Hz eASSR. (**A**) Each individual’s electrically-evoked 40-Hz auditory steady-state response (eASSR) amplitude (vector length) as a function of the response phase. Only eASSRs that were significantly different from the noise are shown. Color coding correspond to the those as used in the individual graphs in (**B**) (N = 9). The solid orange line at the center of the graph and the shaded area show the group’s average neural background noise + one standard deviation. (**B**) Each individual’s 40-Hz eASSR amplitude and neural background noise as a function of modulation depth. Error bars show the 40-Hz eASSR amplitude ± the expected variability of the response (i.e., the level of the neural background noise^42^). Error Bars are only shown in case the response was significantly different from the neural background noise. Figures are made in R^69^.

Stimulation artifacts could be removed by means of linear interpolation in eight out of ten subjects but only for the contralateral recording electrodes. The latency of the ipsilateral response to the CI recording electrodes was 0 ms, indicating that the artifact could not be removed from these channels (not shown). In the following we only report on the recording electrodes positioned contralateral to the CI and referenced to Cz. Figure 2C,D shows the phase and amplitude characteristics of the obtained responses after artifact removal. Linear interpolation, with a maximum duration, was unable to remove the stimulation artifacts in subjects S4 and S6, resulting in a zero slope (i.e. the artifact duration was longer than the interpolation length). The eASSR strength of S4 was just above the noise floor, which was not the case for S6 (Fig. 2C). The average eASSR magnitude of all artifact-free and significant responses was 0.47 µV (SD = 0.41 µV, range = 0.10 to 1.52 µV). The phase-frequency functions of those responses had an average slope of 14.47°/Hz (Fig. 2C). The corresponding latencies were on average 40.2 ms (SD = 8.9 ms), which is similar to those of CI users stimulated with pulse trains around 500 pps^42,43^ and to latencies obtained from normal-hearing listeners^42,43^. Furthermore, the phases of the 40-Hz eASSRs in the modulation depth experiment were, for these eight subjects, consistent across the different MDs_%_ and not centered on a multiple of 180°, i.e. the phase of the stimulation artifact. This was not the case for subject S4 (Fig. 3A), from whose responses the artifact could not be removed. These results show that the 40-Hz eASSRs of these eight subjects were, after artifact removal, true neural responses that are not affected by the stimulation artifacts.

### Effect of modulation depth on electrophysiological responses

**Figure 4:**
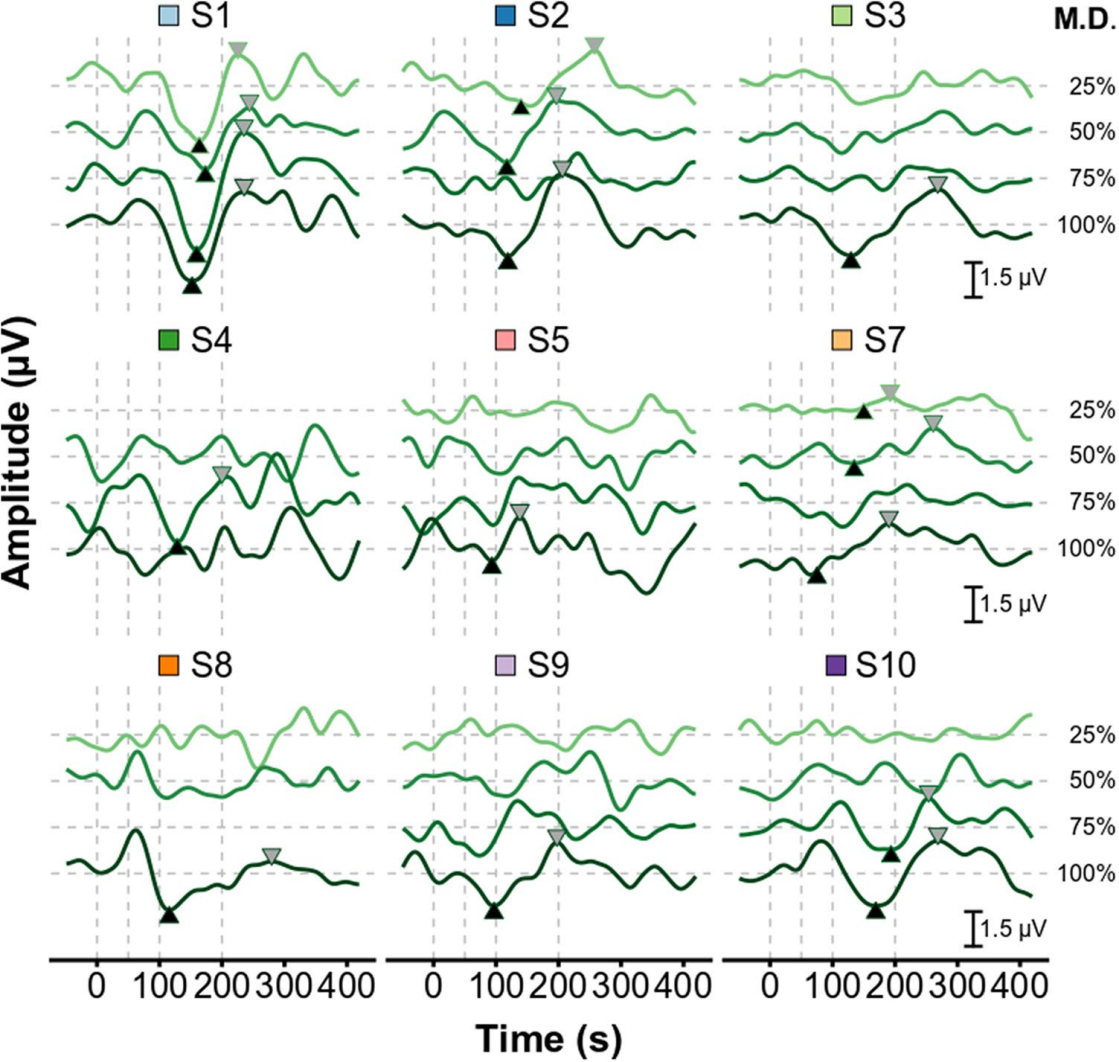
Effect of modulation depth on the eACC waveform. Each individual’s electrically-evoked auditory change complex (eACC) waveforms as a function of modulation depth. Different offsets and colors are used for the different modulation depths (M.D.). N1 and P2 peaks are indicated in case of a significant ACC. Subjects are color coded to facilitate an easy comparison between figures throughout the article. The difference in offset between modulation depths is 2 µV. Figures are made in R^69^.

**Figure 5:**
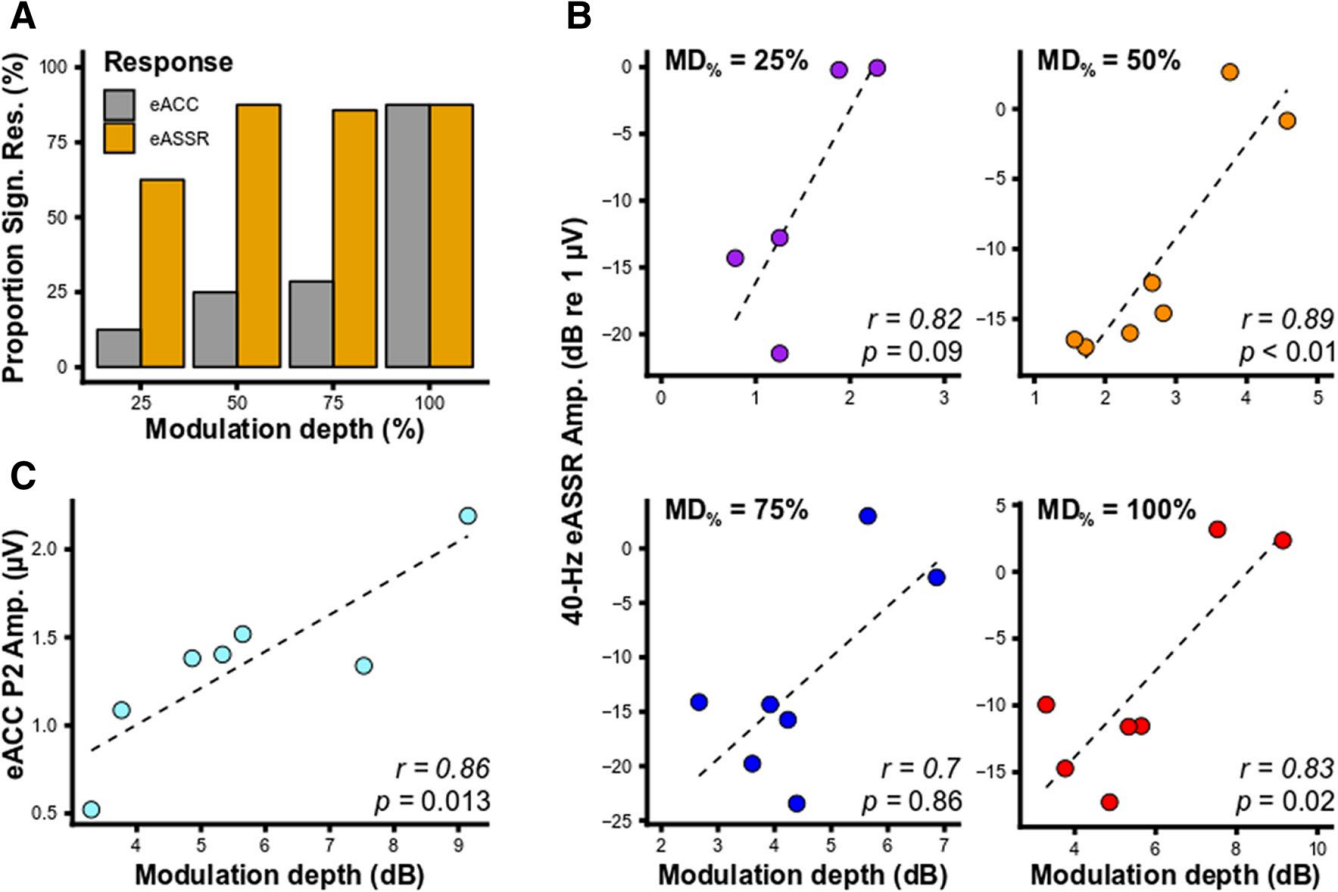
Percentage detected responses and correlations between the modulation depth and the response amplitude. (**A**) The proportion of detected electrophysiological responses as a function of modulation depth. (**B**) Scatterplots showing the 40-Hz electrically-evoked auditory steady-state response (eASSR) amplitude as a function of the modulation depth in dB current for the different modulation depth conditions (MD_%_). (**C**) The electrically-evoked auditory change complex (eACC) P2 amplitude as a function of modulation depth in current for the 100% modulation condition. Only subjects with significant responses are included in (**B**,**C**). Figures are made in R^69^.

Figure 3 and 4 show, respectively, the 40-Hz eASSR and eACC as a function of MD_%_ for each subject. The 40-Hz eASSRs could be evoked in ~ 80% of the subjects for MDs_%_ > 25% (Fig. 5A). 40-Hz eASSR magnitudes varied across subjects and showed either a stable or decreasing pattern with decreasing MD_%_. Although some individual patterns showed a decrease in response strength with decreasing MD_%_, there was no significant group level effect of MD_%_ on the 40-Hz eASSR amplitude as determined by one-way ANOVA (F(3,23) = 0.167 p = 0.918). 40-Hz eASSR magnitudes were on average 0.37 µV (SD = 0.42 µV) for the 25% MD_%_ condition, 0.45 µV (SD = 0.49 µV), for the 50% MD_%_ condition 0.47 µV (SD = 0.52 µV), for the 75% MD_%_ condition, and 0.56 µV (SD = 0.56 µV) for the 100% MD_%_ condition.

The morphology of the eACC varied across participants and conditions (Fig. 4) and eACCs could only be evoked in ~ 80% of the subjects for the 100% MD: for all the other MDs fewer than 30% of the subjects had a significant response (Fig. 5A). N1 magnitude was on average − 1.35 µV (range − 3.1 to 0.15 µV) and its latency was on average 135.9 ms (range 75.0 to 193.5 ms). The P2 magnitude was on average 1.31 µV (range 0.52 to 2.19 µV) and its latency was on average 226.5 ms (range 138.1 to 279.5 ms).

There was no significant across-subject correlation between the ASSR magnitude and the N1 and P2 magnitude or N1 and P2 latency. This indicates that although both measures are affected by the of the MD, they do not covary. Subject S10 for example has, although prolonged latencies, classical ACC waveforms but very low ASSR magnitudes; in contrast, S7 and S8 have ASSR magnitudes within the normal range of those reported in the literature^19,42^ but non-classical ACC waveforms.

### Association between dynamic range and electrophysiological response strength

To investigate if a larger dynamic range results in a better representation of the TEM in the neural code, hence a larger electrophysiological response, we assess whether the modulation depth in dB current (MD_dB_) was associated with the strength of both the 40-Hz eASSR and eACC. We found that MD_dB_ correlated significantly with the ASSR magnitude for the 50% (*r*(5)_pearson_ = 0.89, p = 0.007), and 100% modulation condition (*r*(5)_pearson_ = 0.83, p = 0.021), but not for the 25% (*r*(3)_pearson_ = 0.82, p = 0.091) and 75% modulation condition (*r*(5)_pearson_ = 0.69, p = 0.086) (Fig. 5B). Furthermore, the MD_dB_ of the 100% modulation condition was significantly correlated with the P2 amplitude (*r*(5)_pearson_ = 0.86, p = 0.013) (Fig. 5C), but not with the N1 amplitude (*r*(5)_pearson_ = -0.10, p = 0.83) or the N1 (*r*(5)_pearson_ = 0.19, p = 0.69) and P2 (*r*(5)_pearson_ = -0.9, p = 0.69) latencies. This indicates that both the 40-Hz eASSR magnitude as the P2 amplitude of the ACC are affected by the dynamic range used for stimulation.

### Reliability of the electrophysiological responses

**Figure 6:**
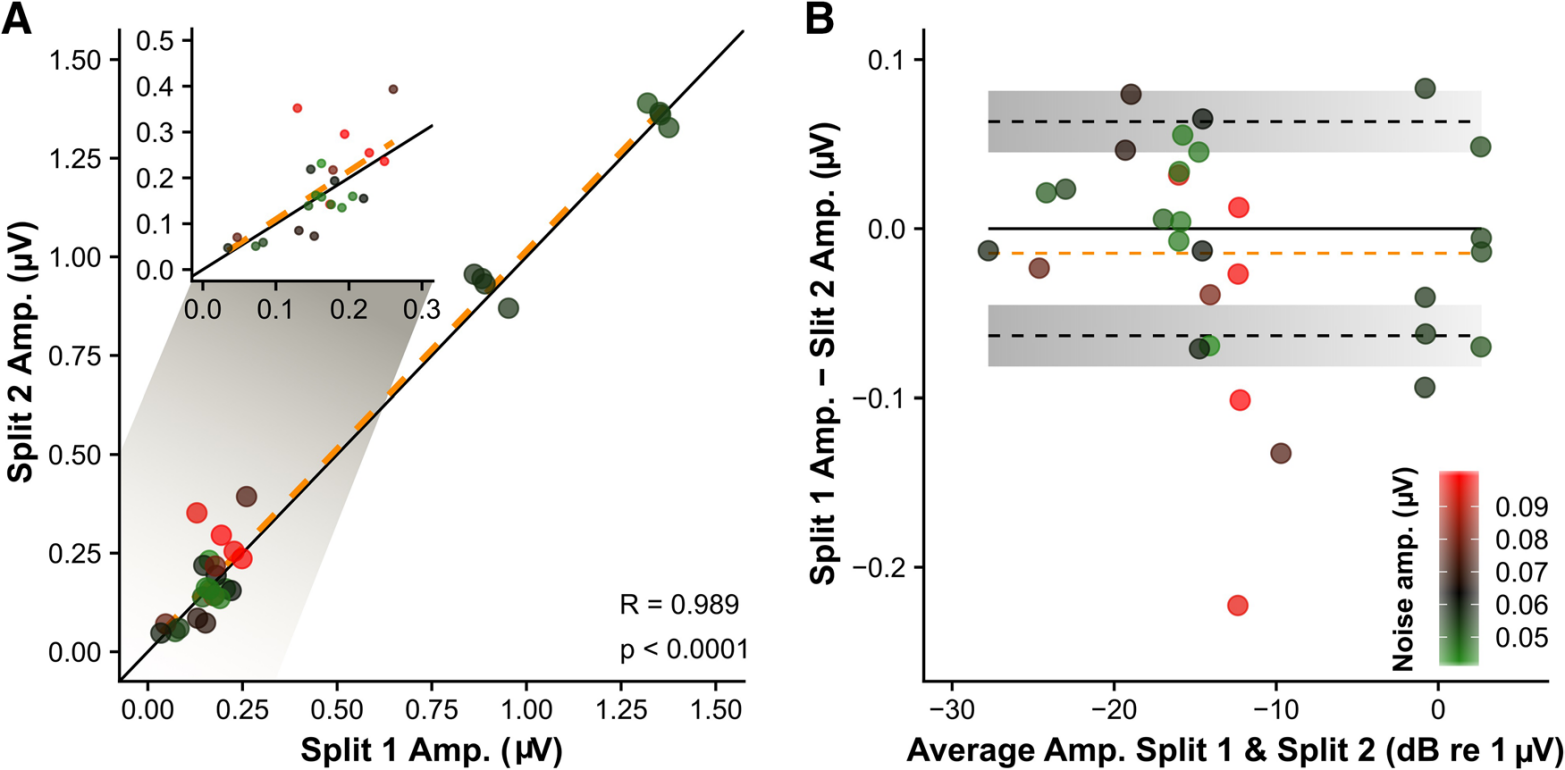
40-Hz eASSR reliability results. (**A**) The 40-Hz eASSR amplitudes of split-recording 2 as a function of the 40-Hz eASSR amplitudes of split-recording 1 across subjects and conditions. The solid black line represents the line of equality and the dashed orange line the linear regression based on all the data. The upper-left figure in (**A**) shows the data within the 0–0.3 µV amplitude range, the regression line is the same as in the main figure. (**B**) The difference in amplitude between the 40-Hz eASSR of split-recording 1 and 2 as a function of the average amplitude across split-recordings. The dashed orange line represents the grand average difference, the dashed black lines shown ± the grand average neural background noise and the grey shaded area grand average neural background noise ± the standard deviation. The color (as indicated by the color bar) in (**A**,**B**) indicates the average neural background noise level across both split-recordings, in which the grand mean background noise (63.3 nV) is shown in black. Figures are made in R^69^.

To gain insight in the reliability of the electrophysiological responses, we randomly divided the recordings of the 40-Hz eASSRs and eACCs into equal unique trials (a split-recording half-half) and found that the 40-Hz eASSR magnitude across all subjects and conditions highly correlated between the split-recordings (*r*(29)_pearson_ = 0.99, p < 0.0001) (Fig. 6A). Furthermore, the difference in magnitude between the split-recordings was within the average across-subjects neural background noise, except for the recordings where the neural background noise of the specific recording exceeded that of the average group neural background level (Fig. 6B). For the eACC we only assessed the MD_%_ 100% condition, since this condition resulted in almost all of the significant responses across subjects. There were only 6 out of 7 eACCs significant in the first split-recording and only 3 out of 7 in the second split-recording, hence further analysis was not possible. These results show that even half of the used trials result in very robust responses for the 40-Hz eASSr. However, the eACC requires more averages. One has to take into account when interpreting these results that the variability in the response magnitude across recordings is directly related to the neural background noise^42^ which normally decreases with a factor equal to the square root of the number of averages. For example, the average, neural background noise for the 40-Hz eASSR across subjects and conditions, increased from 42.7 to 63.3 nV when halving the number of averaged trials. The variability of the obtained responses when all trials are averaged is therefore less than those reported on the split-recordings, indicating that the responses reported here are robust.

## Discussion

Measuring the neural response to continuous electrical stimulation is inevitably corrupted by the large stimulation artifact in the recorded EEG signal, which is not only specific to CIs but also to other biomedical devices^30^. This is especially problematic when the neural response cannot be discriminated from the stimulation artifact, as is the case for eASSRs measured from CI users. The removal of stimulation artifacts from the EEG-recording is challenging especially when they result from stimulating at clinically relevant pulse rates, or higher, and in monopolar stimulation mode^19,24,25,29,45,46^. In order to enable artifact removal—i.e. reducing the artifact waveform distortion and duration—for these clinically relevant stimulation parameters, we used here a newly designed EEG recording system that operates at a sample rate that exceeds those of conventional EEG systems (i.e. at 262 kHz). Our results show that, by using this hyper-rate EEG system and applying linear interpolation, stimulation artifacts could effectively be removed from the EEG recording in the majority of the subjects when recording from locations contralateral to the CI. The maximum pulse rate that effectively can be used to remove the stimulation artifacts, when stimulating in monopolar mode and record the EEG with a conventional sample rate < 16 kHz, was 500 pps^13,19,23,29^. The advantage of the hyper-rate system, as used here, is that it limits the distortion of the artifact waveform^30^ and therefore shortens its duration, and hence enable the use of higher pulse rates when using the linear interpolation method for artifact removal.

Temporal envelope encoding in the auditory pathway is of importance for CI users since these are the main features that are transmitted by the CI^4^. The ability of a CI user to detect modulations in a signal are associated with better speech perception^47,48^. Furthermore, the processing of TEMs can depend on the stimulated neural ensembles, and disabling electrodes that elicit high MDTs has been shown to improve speech perception in noise^12–14^. Given that a large variation in TEMs processing of the neural ensembles located across the CI electrode array can be detrimental to speech perception, there is a potential for improvement based on fitting methods that take this variability into account. In the search of a clinically feasible method to determine TEM encoding we investigated how both the 40-Hz eASSR and the eACC are affected by MD_%_. We found that both the 40-Hz eASSR and eACC are affected by MD_%_ but differently. Whereas the 40-Hz eASSR is only slightly affected by the MD_%_s as used, the eACC could only be elicited by a full modulation in the majority of subjects.

At present, it is unclear if the used MDs_%_ are perceptually different from each other and how this affects the ASSR. Monoghan et al.^49^ for example, found that CI users, based on psychophysics, are insensitive to differences in modulation depth across a large part of the dynamic range. Nevertheless, the relatively stable patterns as a function of modulation depth as reported here have been also observed in normal-hearing humans when using acoustical stimulation^50^. Dimitrijevic et al.^50^ found that there is only a limited growth in ASSR strength with the increase of MD from 50 to 100% and similar results have been reported by Picton et al.^43^. Furthermore, the variation in 40-Hz ASSR as a function of MD is affected by age, with a shallower/absent growth for MDs > 40% with advancing age^50^. The comparison of the eASSR obtained in CI users with the ASSRs as obtained in normal-hearing people has to be interpreted with caution. Loudness growth functions differ markedly between acoustic- and electric stimulation; normal-hearing people have a compressive loudness growth function^51^ whereas CI users have a linear loudness growth^51^ that steeply increases after a specific knee point^41^. The relatively stable patterns as a function of modulation depth as observed here indicate that variations in the 40-Hz eASSR amplitude across the CI array, as reported by Gransier et al.^13^ probably reflect rather large differences in TEM encoding between the stimulated neural ensembles across the CI array, especially since large differences in MD—up to 75% of the dynamic range—did not result into measurable differences in eASSR amplitude in the present study.

Although the 40-Hz ASSR was present for most subjects and MDs, this was not the case for the eACC. Furthermore, there was no association between the eACC and the 40-Hz eASSR. This suggests that the responses reflect different mechanism in the auditory pathway. Whereas the 40-Hz eASSR is a phase-locked response and can only be evoked when there is phase-locking to the temporal envelope, the eACC is elicited by a change in stimulus characteristics. The ACC strength, evoked acoustically in the normal-hearing auditory pathway, has been shown to decrease with decreasing MD^32^ and has the same lowpass characteristic as the behavioral temporal modulation transfer function^32^. In contrast, the findings in the acoustically stimulated normal-hearing auditory pathway are not straightforward to translate to the eACCs elicited in the electrically stimulated deafened auditory pathway. One assumes, when acoustically stimulating the normal-hearing auditory pathway, that the mechanisms involved in processing TEMs are relatively equal across subjects. Therefore, by controlling for all other factors that could evoke an ACC—e.g. loudness and pitch—one can assess the ACC as a function of MD. However, this translation is not so straightforward in the case of CI recipients. When interpreting the eACC one has to consider that reduced or absent phase-locking can result in a neural signal that the brain interprets as different from an unmodulated signal, and that this difference can evoke an eACC. The eACC can thus be evoked when the neural encoding of the two stimuli is different, it does, however, not mean that the TEM is represented properly in the neural code, hence an absent or reduced 40-Hz eASSR. S1 and S10 are an example of such a potential effect. S1 has both 40-Hz eASSRs and ACC, whereas S10 has very small or absent 40-Hz eASSRs but eACCs that are about as large as those of S1. Furthermore, one has to consider that at an early stage loudness integration occurs at a timescale of about 7 ms^52^ compared to the overall-loudness perception which is typically 300 ms^53^ as assessed with the loudness balancing task used. In addition, eACCs have been measured to gaps as short as 5 ms^54^. It is therefore difficult to determine based on the protocol used if the eACCs measured were due to the first valley in the modulation or to the processing of several cycles of modulation, since both become less detectible with decreasing MD. Although most subjects reported that they heard the modulation during the experiment, it is not clear what triggered the eACC. Nevertheless, Mathew et al.^35,36^ successfully used the eACC to assess electrode discrimination, and as long as the alteration made between two alternating stimuli cannot be attributed to confounding factors, this can be a clinically relevant measure which is little troubled by the stimulation artifacts.

The electrophysiological measures used in the present study originate from regions in the auditory pathway beyond the periphery^17,38^ and are assumed to reflect the processing cascade of all neural ensembles involved in TEM encoding of the ascending auditory pathway up to generator of the neural response. It is therefore difficult to derive from these measures where in the ascending auditory pathway TEM encoding is affected. An important bottleneck in electrical stimulation of the auditory pathway is the electrode-neuron interface^55^ and there is indirect evidence that measures like the 40-Hz eASSR but also the MDTs as assessed behaviorally reflect the TEM encoding ability at the level of the electrode-neuron interface^11,13^. Electrophysiological measures from the periphery could potentially be complementary to the more central measures and can provide insight where in the auditory pathway TEM encoding is affected. Studies have shown that ECAPs can be obtained to each pulse of a modulated pulse trains both in animals^56^ and humans^57^. Jeng et al.^56^ found that the electrically-evoked compound action potential (eCAP) modulated response amplitudes (i.e. difference between the minimum and maximum eCAP amplitudes across a modulated cycle) show a compressive non-linear growth with increasing modulation depths, which is in line with the 40-Hz eASSR growth functions that we observed. Furthermore, Tejani et al.^57^ measured the eCAPs to AM pulse trains with high modulation rates (> 100 Hz) and found that the slope of the ECAP slope as a function of current used in the AM pulse train was correlated with the MDTs of the same stimuli. The combination of peripheral and more central electrophysiological measures can potentially provide more insight in the location of TEM encoding deficits in the electrically-stimulated auditory pathway.

Our results could, in addition to the assessment of TEM processing, potentially pave the way for the objective assessment of detection thresholds in CI users. Threshold determination is of importance for programming the CI sound processor for clinical use^4^. Nevertheless, this is challenging in CI recipients who are unable to give reliable subjective feedback, such as infants, young children, and adults who are intellectually challenged. Detection thresholds are, however, known to decrease with increasing pulse rate due to multi-pulse integration. The slope of this decrease as a function of pulse rate can be highly variable across subjects and stimulation sites^11,58,59^, and is associated with the neural health of the stimulated neural ensembles^60^. This makes it difficult to estimate the stimulation thresholds in CI users based on electrophysiologic measures that use low-rate stimuli, such as the eCAP and eABR^58^. The advantage of the eASSR is that one can modulate a high-rate carrier to obtain the electrophysiological response, and hence obtain the electrophysiological thresholds with the same pulse rate as used in the clinical device. Hofmann and Wouters^22^ and Van Eeckhoutte et al.^61^ both measured the 40-Hz eASSR magnitude as a function of current in bipolar mode and reported that the growth functions corresponded with loudness perception^61^ and that that the detection thresholds could be estimated from the growth functions^22^. Our results show that the 40-Hz eASSR can be obtained, free from stimulation artifacts when using a clinically-relevant pulse rate of 900 pps and monopolar stimulation. Furthermore, our results show a relative flat 40-Hz eASSR magnitude as a function of modulation depth, indicating that when using a modulation depth that corresponds to the dynamic range of the average CI user, one should be able to evoke 40-Hz eASSRs in most CI users without a large reduction of the response strength compared to using the maximal perceptual modulation depth. Taking this into account and given that ASSRs are used clinically to determine hearing thresholds of infants with a suspicion of hearing loss^21^, it should be feasible to determine detection thresholds to clinically-relevant stimulation pulse rates, stimulated in monopolar mode, by the means of the 40-Hz eASSR in adult CI users, when using the hyper-rate EEG system to record the EEG and linear interpolation for artifact removal.

## Methods

### Stimulation

All stimuli were generated in Matlab R2016b and were delivered directly to the implant by the means of a research interface, which consisted of a laptop with custom-written software interfacing with the Nucleus Implant Communicator (version 3) and connected to the implant through a programming device and an L34 research processor. The hardware and the Nucleus Implant Communicator were provided by Cochlear Ltd. The pulse trains used in all experiments were either unmodulated or amplitude modulated and consisted of symmetric biphasic cathodic-first pulses, with a phase width of 25 µs and an interphase gap of 8 µs. Pulse trains were presented at 900 pps to the most apical electrode (e22) in monopolar mode (i.e. both the extracochlear electrode on the casing and the extracochlear ball electrode were used as the return electrodes). The most apical electrode was chosen based on the results of Gransier et al.^13^ They evoked a 40-Hz eASSR with each stimulation electrode of the electrode array separately and found that, although the 40-Hz eASSR could be evoked in almost all CI users and from all CI electrodes, the most apical electrode had a variable 40-Hz eASSR amplitude across CI users. Furthermore, Luke et al.^20^ found that the 40-Hz eASSR amplitude is associated with the behavioral MDT (i.e. larger 40-Hz eASSR amplitudes correspond to better MDTs). Based on these results we considered the most apical stimulation electrode to be ideal to assess the association between the 40-Hz eASSR and the eACC.

### Behavioral assessment of stimulation levels

Stimulation levels were determined behaviorally at the beginning of each experiment by using a 7-point categorical loudness scale (i.e. “inaudible”, “very soft”, “comfortably loud”, “loud”, “very loud”, and “unbearable”). First, the maximum comfort level for the unmodulated pulse train (C_unmod_) was assessed by starting ~ 20 current units, which corresponds to roughly 3 dB below the threshold level of the participant’s clinical map, and the current was then gradually increased to obtain the most comfortable loudness level which is defined the current level prior to the transition between “comfortably loud” and “loud” on the categorical loudness scale. Second, the current was gradually decreased to determine the threshold level of the unmodulated pulse train (T_unmod_). T_unmod_ was defined as the current level below which the participant could not perceive the stimulus anymore. Third, the comfort level of the 40-Hz 100% amplitude modulated stimulus (C_mod_) was determined. The minimum of the modulation was fixed at T_unmod_ and current of the other pulses in the sequence were increased until C_mod_ was achieved. C_mod_ was determined the same way as C_unmod_. The amplitude modulation of all modulated stimuli was sinusoidal on a linear current scale. Although, the behavioral levels were initially assessed in current levels, the currents reported here are in dB re 1 µA. Equation 1 and 2, as provided by Cochlear ltd, were used to calculate the current in dB re 1 µA from the current levels—as send to the programming device—for the CIC3 and CIC4 implants, respectively.

1 $$I\left( {\mu A} \right) = 10 \times e^{{\frac{{CL \cdot \ln \left( {175} \right)}}{255}}}$$

2 $$I\left( {\mu A} \right) = 17.5 \times 100^{{\frac{CL}{{255}}}}$$

MDs (25, 50, 75, and 100%) were based on the dynamic range (i.e. 100% MD), which is defined as the difference between C_mod_ and T_unmod_. The different MDs were based on the current (in dB). To minimize the effect of overall loudness differences between the unmodulated and modulated pulse trains on the ACC, the modulated pulse trains were loudness-balanced to the unmodulated pulse train. This procedure was done for each modulation depth separately. During the loudness-balancing task, a two-down, one-up procedure was used and the participant had to indicate which presentation was louder (either the unmodulated or the modulated stimulus) in a two-alternative forced choice paradigm. Step sizes of one or two current units were used depending on the discrimination ability of the participant and the MD was kept constant across trials. The loudness balancing procedure was stopped after eight reversals and the mean of the last six reversals was used as the loudness balanced level.

### Electrophysiological measures

The electrophysiological measurements consisted of two parts. First, we recorded the EEG to amplitude-modulated pulse trains with modulation frequencies of 34, 37, 40, and 43 Hz. This was done to evaluate the effectiveness of the offline-applied, artifact removal method. The rationale behind these measurements is that the group delay of the generator(s) from which the ASSR originate is constant for closely spaced modulation frequencies^42,43^. When the artifact is effectively removed and/or the neural response dominates the EEG recording a group delay around 40 ms is expected^19,42^, whereas a group delay of 0 ms expected if the recordings are dominated by the stimulation artifact^19,22^. In the following we refer to this part of the EEG experiment as the *group delay experiment*. We measured the EEG to the different modulated pulse trains at a 100% MD. This MD was chosen since the artifact component at the modulation frequency increases with increasing MD, so by using a 100% modulation we were able to evaluate artifact removal for the most extreme condition per subject. Stimulation levels were subject-specific and were those that were determined during the behavioral assessment of the stimulation levels. The stimulus consisted of 300 epochs each with a duration of 1.024 s, and modulation frequencies and pulse rates were adjusted so that each epoch included an integer number of cycles/pulses, only the rounded modulation frequencies/pulse rates are reported. A trigger was sent at the start of each epoch from the programming device to synchronize the stimulation and recording.

Second, we recorded the EEG to the stimuli that were used to assess the effect of MD on the 40-Hz ASSR and the ACC. A single trial consisted of a 2.024 s unmodulated pulse train and a 2.024 s 40-Hz AM pulse train. The MD of the modulated part was changed across conditions (i.e. 25, 50, 75, and 100%), and the stimulation levels were set to those as determined during the behavioral assessment of the stimulation levels. 100 trials of a specific MD were concatenated and presented in one block, and a trigger was sent at the start of each trial from the programming device to synchronize the stimulation and recording. Two blocks of each MD were presented in random order during a session. Subject S6 perceived the loudness balanced levels as too loud during the EEG experiments. Although stimulation levels were lowered and the EEG experiment was carried out, she was excluded from the analysis since the results were potentially cofounded by loudness cues. For this reason, S6 is only included in the analysis of the first EEG experiment in the “Results” section. In the following we refer to this part of the EEG experiment as the *modulation depth experiment.*

EEG was recorded with an 8-channel Biosemi ActiveTwo Hyper-Rate EEG recording system designed and built to our specifications. This system is based on the Biosemi ActiveTwo EEG-recording system but with a sample rate of 262.144 kHz/channel and with a built-in analog third-order antialiasing filter having a −3 dB point at 50 kHz. We used Ag/AgCl active recording electrodes to record the EEG. The recording electrodes were placed at the subject’s head using an 8-channel cap according to a 10–20 system^62^ layout. The position of the recording electrodes on the ipsilateral to the CI site differed across subjects due to the location of the CI, but were always placed in a close proximity to the mastoid, if possible. Individual differences in placement were the result of the location of the RF receiver of the CI, since no recording electrodes could be placed there due to the presence of the research sound processor. The results reported are based on recording electrodes that are placed in close proximity of the left and right mastoid (the recording electrodes contralateral to the CI were always P10 or P9) and referenced to Cz. This electrode configuration was chosen since it optimally captures the electrical potential originating from 40-Hz ASSR generator(s)^63^.

All EEG recordings were made in a Faraday cage, an electrically shielded sound booth, where participants sat in a comfortable chair. To minimize tension in the muscles supporting the head, the head and neck were supported by the chair and occasionally by a cushion. To reduce artifacts caused by movements, participants were asked to move as little as possible during the recording. A silent movie with subtitles was played to ensure that the attentional state was similar in all conditions and for all participants. This approach was identical to that of Gransier et al.^13,19^.

### Analysis

All off-line signal processing was done in Matlab (version 2016b)^64^. CI-stimulation artifacts were first removed from the raw EEG signal by means of linear interpolation between a prestimulation pulse sample and a poststimulation pulse sample. The prestimulation pulse sample was set at 100 µs and the poststimulation pulse sample at 900 µs after the start of each stimulation pulse. After linear interpolation, the time signal of each recording electrode was high-pass filtered using a 2nd order Butterworth high-pass filter with a cutoff frequency of 2 Hz, to remove any DC component in the recordings.

For the ASSR analysis we divided each time signal of each recording into individual epochs, each with a length of 1.024 s for the *group delay experiment* and 2.048 s for the *modulation depth experiment*, based on the triggers. The EEG response to the unmodulated part of each trial in the *modulation depth experiment* was omitted from the analysis. Epochs of the different recording blocks in the *modulation depth experiment* were combined, resulting in a total of 200 epochs per MD. Five percent of the total number of epochs with the highest peak to peak amplitude were removed from the recordings to minimize the effect of the physiologic and extraphysiologic artifacts containing recording epochs. A Fast Fourier Transform was used to calculate the complex frequency spectrum of each epoch, resulting in a frequency resolution of 0.98 and 0.49 Hz for the *group delay* and *modulation depth experiment*, respectively. To remove the common noise across electrodes and to optimally capture the dipoles originating from the 40-Hz ASSR generator(s), the different channels and electrodes were referenced to the electrode positioned at Cz. Referencing was done by subtracting the complex frequency spectrum of the reference electrode from the complex frequency spectrum of each channel and the recording electrode. To compensate for the filter effects on the magnitude of the response, the inverse gain of the high-pass filter was applied to the frequency spectrum of each epoch. For each epoch, the response power, amplitudes, and phases were obtained from the complex frequency spectrum corresponding to the modulation frequencies used during the experiment (i.e. the response spectrum). The mean response amplitude and phase were computed by vector averaging the complex response spectrum across epochs. The neural background noise was calculated as the standard deviation over epochs divided by the square root of the number of epochs^42^. The Hotelling T^2^ was used, for each channel, to determine whether the synchronized activity (i.e. the measured response) differed significantly from the nonsynchronized neural background activity. This test compares the average real and imaginary components of the response spectrum with the variability across epochs of the response spectrum^22,65^. We derived the group delay (i.e. the latency of the generator) in the *group delay experiment* from the response phase slope across the modulation frequencies.

For the ACC analysis, we referenced the raw-EEG time signals after blanking to Cz and divided each referenced time signal into individual trials based on the triggers. Trials of the different recording blocks were combined, resulting in a total of 200 trials per MD. Five percent of the total number of trials with the highest peak to peak amplitude were removed from the recordings to minimize the effect of the physiologic and extraphysiologic artifacts containing recording trials. A low-pass filter (eegfilt, from EEGLAB) with a cutoff frequency of 14 Hz was then applied for analyzing the ACCs to the change from the constant to the modulated part of the trial. The Golding et al.^66^ implementation of the Hotelling T^2^ test was used to determine if an ACC was present, and peaks and latencies were determined using a semi-automatic procedure. We then analyzed the N1 and P2 peak of the ACC separately since both are assumed to originate from different generators and are associated with different auditory processes^67,68^. All other statistical analyses and figures were done in R (version 3.5.3)^69^ and a significance level of 5% was used.

### Participant information

Ten adult native Flemish-Dutch-speaking CI users took part. All had a history of long-term hearing impairment with an average CI use of 4.3 years. All participants had a device from Cochlear Ltd. and were followed up at the ear, nose, throat clinic and the Multidisciplinary Center for Logopedics and Audiology (MUCLA) of the University Hospital in Leuven, Belgium. Participant details are shown in Table 1. This study was approved by the Medical Ethics Committee of the University Hospital in Leuven (Approval number: B322201524931) and all methods were carried out in accordance with the relevant guidelines and regulations. Written informed consent was obtained from all participants before testing.

**Table 1:** Participant details.

Subject	Age (years)	Gender	CI	CI side	Duration CI use (months)
S1	68	Female	CI24RE	Right	83
S2	28	Female	CI24R	Left	168
S3	76	Female	CI522	Left	13
S4	58	Female	CI522	Right	41
S5	63	Female	CI522	Right	16
S6	48	Female	CI422	Right	63
S7	40	Female	CI522	Left	17
S8	64	Female	CI5222	Right	11
S9	57	Male	CI24RE	Right	96
S10	59	Female	CI522	Right	7
